# Biliary cystadenocarcinoma of the gall bladder: a case report

**DOI:** 10.1186/1752-1947-3-75

**Published:** 2009-10-15

**Authors:** Sarath Chandra Sistla, Gomati Sankar, Debadutta Basu, Bhuvaneswari Venkatesan

**Affiliations:** 1 Department of Surgery, Jawaharlal Institute of Postgraduate Medical Education and Research, Pondicherry, India; 2 Department of Pathology, Jawaharlal Institute of Postgraduate Medical Education and Research, Pondicherry, India; 3 Department of Radio Diagnosis, Jawaharlal Institute of Postgraduate Medical Education and Research, Pondicherry, India

## Abstract

**Introduction:**

While biliary cystadenoma and biliary cystadenocarcinoma involving the liver are not uncommon, biliary cystadenocarcinoma of the gall bladder is an extremely rare lesion and can be very difficult to diagnose.

**Case presentation:**

A 50-year-old Indian woman presented with pain and swelling in the right hypochondrium. An ultrasonography revealed a cystic lesion arising from the gallbladder fossa. This lesion was initially managed with aspiration and antibiotics by the treating physician. The patient was referred for surgical management because the abscess was not resolved through conservative treatment. A diagnosis of an infected nonparasitic cyst was made and deroofing of the cyst was performed. A histopathological examination of the excised cyst wall showed cystadenocarcinoma. The patient subsequently underwent a successful surgical excision of the lesion.

**Conclusion:**

Infective lesions of the liver are common in developing countries and are usually managed through aspiration and antibiotics. Cystadenocarcinoma of the gallbladder needs to be considered in the differential diagnosis of cystic lesions arising from the gallbladder fossa. A high index of suspicion and cytological examination from the wall of such complex lesions will help in the timely management of such lesions.

## Introduction

Extra hepatic biliary cystadenocarcinomas are uncommon and most often arise from the hepatic ducts. Cystadenocarcinomas arising from the gallbladder are extremely rare [1-3]. These lesions pose a diagnostic challenge and need to be differentiated from other more common cystic lesions of the liver such as simple cysts, parasitic cysts and abscesses. We describe the clinical, radiological and histological features of a case of biliary cystadenocarcinoma of the gallbladder, which was successfully managed with surgery.

## Case presentation

**Figure 1:**
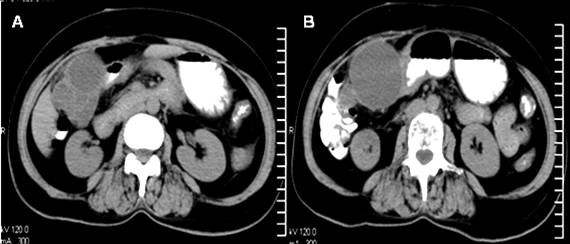
**A computed tomography scan image showing the multilocular cyst with septations (a) and its proximity to the duodenum and colon (b)**.

**Figure 2:**
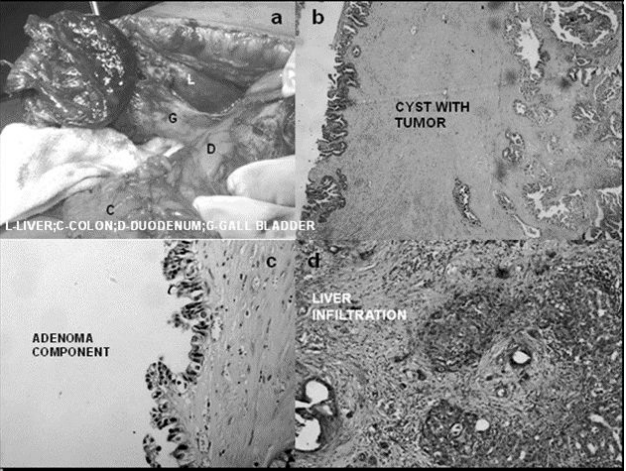
**An operative image (a), a cyst wall with tumor H&E ×100 (b), area showing adenoma H&E ×200 (c) and liver infiltration by the tumor H&E 100 (d)**.

A 50-year-old Indian woman with diabetes and hypertension presented with right upper abdominal discomfort, loss of appetite and low grade fever. She had no history of jaundice. Examination revealed hepatomegaly and tenderness in the right hypochondrium. An abdominal sonography revealed an 11 × 7.5 × 11.2 cm, predominantly anechoic, thick-walled lesion arising from the inferior surface of the liver with a few low-level internal echoes and a homogenous echogenic area in the superolateral portion of the lesion. The gallbladder could not be visualized separately and the other abdominal viscera were normal. Her liver function tests were normal and serological tests for amoebiasis and hydatid disease were negative. Her chest X-ray was normal. A diagnosis of liver abscess was made and aspiration of the lesion revealed turbid fluid. However, the lesion did not resolve with repeated aspirations and antibiotics. A contrast enhanced computerized tomography (CECT) scan revealed a well-defined, lobulated cystic lesion with a solid component in the superior part of the lesion. Figure 1a and Figure 1b show the proximity of the lesion to the colon and duodenum. The lesion was interpreted as an infected non-parasitic cyst and evacuation and deroofing of the cyst were performed. Histopathological examination of the excised cyst wall revealed a biliary cystadenoma with nuclear atypia. Subsequently, a radical excision of the lesion with a partial excision of the liver was performed (Figure 2a). The final histological diagnosis was biliary cystadenocarcinoma infiltrating liver (Figure 2b, c, d).

## Discussion

Cystic lesions originating from the gallbladder fossa can be either from the inferior surface of the liver or from the gallbladder and the distinction can sometimes be difficult. In this case, the lesion arose from the gallbladder fossa and the gallbladder was not visualised separately on imaging. It was presumed that the gallbladder was either compressed or displaced by the cystic lesion.

Cystic lesions of hepatic origin, like simple cysts, parasitic cysts and abscesses, are more frequent than those arising from the gallbladder.

A primary hydatid cyst, lymphangioma and an epithelial cyst associated with adenocarcinoma of the gallbladder are some of the cystic lesions of the gallbladder reported in the literature [4-7]. There are few case reports of biliary cystadenoma and cystadenocarcinoma of the gallbladder [1-3]. The origin of these lesions from the gallbladder can be confirmed by either an endoscopic retrograde cholangiopancreaticography (ERCP) to demonstrate communication with the cystic duct, or an angiography to demonstrate the blood supply from a cystic artery.

Because biliary cystadenomas and cystadenocarcinomas are rare, they are not easy to diagnose preoperatively and cannot be accurately distinguished from each other merely by imaging. Simple cysts can usually be differentiated from cystic neoplasms by imaging. Thin, regular, non-enhancing wall and septations are features of simple cysts. However, complications like infection and hemorrhaging make the distinction more difficult. Cystic neoplasms can be misinterpreted as infective lesions of the liver, especially in areas where such lesions are more common, as in the present case. Yu *et al*. reported such a problem in managing hepatic biliary cystadenomas and carcinomas, one of which was initially treated as a liver abscess [8]. While some consider mural nodules to be a feature of cystadenocarcinomas, others have found them both in biliary cystadenomas and cystadenocarcinomas [9,10]. Pojchamarnwiputh *et al*. observed enhanced mural nodules on computed tomographies in all cases of biliary cystadenocarcinoma, but not in patients with cystadenomas [11]. Thick and coarse calcification was seen on CT scans of malignant lesions compared with thin calcification in benign biliary cystic neoplasms [10].

Guided fine needle aspiration cytology (FNAC) from the irregular area of the wall or the mural nodule prior to the first surgery in our patient may have avoided delays. However, definite diagnoses of FNAC of cystic lesions can be difficult owing to poor cellularity, degenerative changes in the cells and reactive changes. Sampling errors are also a problem in these predominantly cystic lesions. Fluid cytology was not found to be useful in detecting malignancy in biliary cystic neoplasms [12].

Elevated levels of carcinoembryonic antigen and CA 19-9 in the cyst fluid were reported [13]. These tumor markers in the serum or cyst fluid, however, do not help in differentiating benign from malignant lesions [12].

Two distinct types of biliary cystadenocarcinomas of the liver are described. The less aggressive tumors with 'ovarian-like stroma' occur in women and the more aggressive type without such stroma are seen in men [14]. Because cystadenocarcinomas are rare, the clinical significance of a lack of ovarian stroma in women is difficult to assess. Our patient's tumor did not show any ovarian stroma.

The gallbladder histology in our case showed a thickened cyst wall lined by a single layer of tall columnar non-mucin secreting cells, focal nuclear pleomorphism and multilayering. Invagination into the underlying subepithelium was also seen. The underlying stroma showed dense fibrosis and congested blood vessels. Following radical excision, a subsequent biopsy of the gallbladder showed a biliary cystadenoma component with a solid area of well-differentiated adenocarcinoma suggesting the malignant transformation of a pre-existing benign lesion.

The tumor in this case could be dissected off the colon and duodenum and we performed a cholecystectomy with a partial resection of the liver. A multivisceral resection was required in the case described by Waldmann [3].

## Conclusion

This case is reported to emphasize that cystadenocarcinoma of the gall bladder needs to be considered in the differential diagnoses of cystic lesions arising from gall bladder fossa. Image guided FNAC from suspicious areas of the wall of the cystic lesions or mural nodules may help in the diagnosis and immediate treatment, as they can be mistaken for infective lesions, in areas where such lesions are common.

## List of abbreviations

CECT: contrast enhanced computerized tomography; ERCP: endoscopic retrograde cholangiopancreaticography; FNAC: fine needle aspiration cytology.

## Competing interests

The authors declare that they have no competing interests.

## Authors' contributions

SCS and GS collected and analyzed the patient's data. SCS and GS prepared the manuscript. DB performed the histological examination. VB interpreted the CT scan images. All the authors read and approved the final manuscript.

## Consent

Written informed consent was obtained from the patient for publication of this case report and accompanying images. A copy of the written consent is available for review by the Editor-in-Chief of this journal
